# Deregulation of Circular RNAs in Cancer From the Perspectives of Aberrant Biogenesis, Transport and Removal

**DOI:** 10.3389/fgene.2019.00016

**Published:** 2019-02-01

**Authors:** Qiongqiong Wu, Peiyao Li, Minghua Wu, Qiang Liu

**Affiliations:** ^1^Hunan Provincial Tumor Hospital and the Affiliated Tumor Hospital of Xiangya Medical School, Central South University, Changsha, China; ^2^Key Laboratory of Carcinogenesis and Cancer Invasion, Chinese Ministry of Education, Cancer Research Institute, Central South University, Changsha, China; ^3^Third Xiangya Hospital, Central South University, Changsha, China

**Keywords:** circRNA, deregulation, cancer, non-coding RNA, genetic

## Abstract

CircRNAs (circular RNAs) are a class of RNAs generated from circularization with multiple novel functions. Recent studies have revealed the aberrant expression and aberrant functions of circRNAs in various tumors; thus, circRNAs have been recognized as promising cancer biomarkers. However, the underlying mechanisms behind their aberrant expression and functions remain unclear. In this review, we discuss at length the cancer-specific deregulation of circRNAs and the potential underlying aberrant events in circRNA biogenesis, localization and removal in cancer cells.

## Background

CircRNAs are a type of RNA in eukaryotes that are spared from exonucleolytic degradation by RNase R given their circular structures and subsequent lack of accessibility of RNase to 3′ and 5′ ends. In 1976, circRNAs were first discovered in a viroid, and they were considered insignificant byproducts for a long period of time (Sanger et al., 1976). Until the last decade, rapid advances in RNA-sequencing have promoted investigations into circular RNAs. CircRNAs can be generally divided into five categories: exonic circRNAs (ecircRNA), circular RNAs from introns (ciRNAs), exon-intron circRNAs (EIciRNA), intergenic circRNAs, and antisense circRNAs (Memczak et al., 2013; Qian et al., 2018). EcircRNAs containing exclusively exon(s) represent the major class, accounting for approximately 85% of all types of circRNAs (Qian et al., 2018). CiRNAs are generated from intron lariats depending on two specific RNA motifs at specific sites, but so far ciRNAs haven’t been revealed to be involved in cancer (Zhang et al., 2013). EIciRNAs consist of both exons and introns that typically localize and function in the nucleus (Li Z. et al., 2015). Two additional circRNAs, namely intergenic circRNAs and antisense circRNAs are not common and are not fully understood to date (Qian et al., 2018). Different types of circRNAs are generated from pre-mRNAs (precursor mRNAs) via different mechanisms (Chen and Yang, 2015; Zhang Y. et al., 2016), such as exon skipping, intron pairing and RNA-binding proteins, that combine to drive the head-to-tail junctions to join together as previously reviewed (Dragomir and Calin, 2018).

In this review, we focus on the new advances in the abnormal expression and functions of circRNAs in cancers, which may account for tumorigenesis and progression. An increasing number of studies have emerged to reveal how circRNAs alter the behavior of tumor cells, but there are no reports on the mechanisms responsible for their abnormal expression. Based on recent findings on circRNAs, we discuss possible mechanisms behind the deregulation of circRNA in cancers to provide insights into the etiology, diagnosis and therapy of cancers.

## Aberrant Expression and Functions of Circrnas in Cancer

In normal tissues, the expression of circRNA exhibits the following characteristics: (1) Conservation of circRNA expression. A study comparing expression patterns of circRNAs among species suggested ancient and conserved features of circRNA expression. The expression of circRNA isoforms is likely to be derived from orthologous genes, and the functional sequence elements of circRNAs are conserved in subsets (Wang P.L. et al., 2014; Barrett and Salzman, 2016; Dong and Ma, 2017). (2) The complexity of circRNA expression (Li X. et al., 2018). A previous study concluded that during evolution, the circRNA expression pattern becomes increasingly complex as the distribution of orientation-opposite complementary sequences in their flanking introns becomes increasingly diverse (Dong and Ma, 2017). From the perspective of individual genes, various circRNAs can be generated from one sequence (Gao et al., 2016). (3) Cell/tissue-specific expression (Zhang Y. et al., 2016). CircRNAs are extraordinarily abundant and diverse in the brain compared with other tissues, and their expression in brain tissue is increased several fold compared with their linear isoforms (Rybak-Wolf et al., 2015). Host genes coding synaptic proteins may serve as a source of abundant circRNA. Recent work in human hematopoietic cells reveals a circular RNA cell-type specific expression pattern (Nicolet et al., 2018). (4) Stage-specific expression. Dynamic expression of certain circRNAs has been observed in some specific developmental stages, such as human pre-implantation embryos (Dang et al., 2016), human fetal development (Szabo et al., 2015), and aging (Westholm et al., 2014). During the differentiation of cells, such as neural cells and myoblasts, abrupt fluctuation of circRNA expression has been reported (Salzman et al., 2013).

The aberrant expression of circRNAs is prevalent in a large number of diseases, especially tumors (Haque and Harries, 2017; Lei et al., 2018; E et al., 2018). As a result, circRNAs have been proposed as biomarkers of diagnosis, prognosis or therapy in specific cancers (Meng et al., 2017; Qian et al., 2018; Wang D. et al., 2018; Yang and Wang, 2018; Zhou J. et al., 2018) based on the convenience of detecting circRNA in the blood plasma of patients. The abnormal expression of circRNAs in cancer is usually accompanied by abnormal functions (Bachmayr-Heyda et al., 2015; Patop and Kadener, 2018).

### Abnormal circRNA/lncRNA/miRNA/mRNA Loop

**FIGURE 1:**
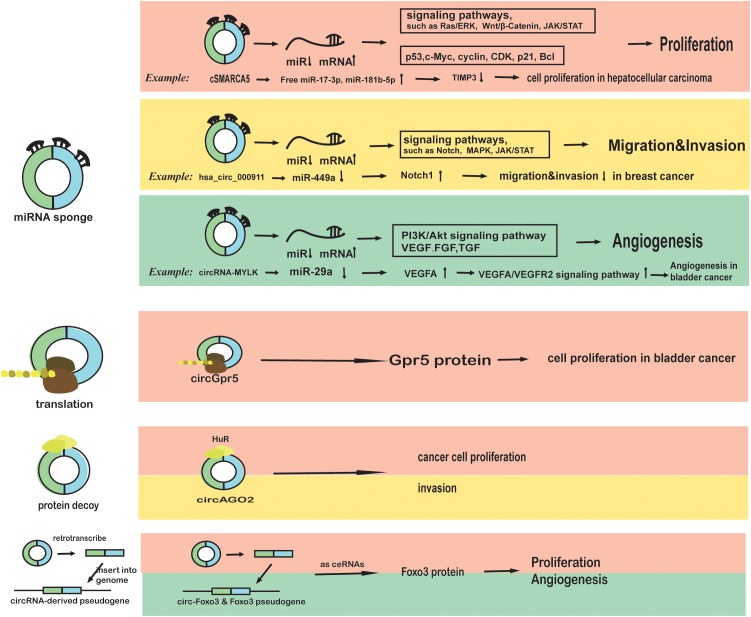
The roles of circRNAs in cancer phenotypes and biological characteristics (proliferation, migration, invasion, and angiogenesis). First, circRNAs may function as miRNA sponges in cancer cells. cSMARCA5 binds to miR-17-3p and miR-181b-5p to inhibit the proliferation of hepatocellular carcinoma cells, which can be blocked by DHX9 (Yu et al., 2018). CircRNA000911 binds to miR-449a to promote migration and invasion by targeting Notch1 and nuclear factor-κB (NF-κB) signaling (Wang H. et al., 2018). CircRNA-MYLK binds to miR-29a and activates VEGFA/VEGFR2 pathway, promoting angiogenesis in bladder cancer (Zhong Z. et al., 2017). Second, circRNAs may be translated in cancer cells. CircGpr5 encodes a peptide that interacts with Gprc5a and circGpr5 to promote bladder cancer (Gu et al., 2018). Third, circRNAs can bind to proteins or function as protein decoys in cancer cells. CircAGO2 can bind to HuR to drive cancer progression (Chen Y. et al., 2018). Fourth, some circRNAs such as circ-Foxo3 can be retro-transcribed and inserted back to the genome to function as competitive RNA to disrupt the function of miRNAs (Yang et al., 2016).

A circRNA called Cdr1as was first discovered as a “miRNA sponge” in human and mouse brains in 2013 (Hansen et al., 2013). CircRNAs, as competitive endogenous RNAs (ceRNAs) with linear mRNAs binding to miRNAs, enhance the expression of target genes (Thomson and Dinger, 2016) and affect the biological behaviors of multiple tumors. For example, the hsa_circ_0007534/miR-761/ZIC5 axis promotes glioma by promoting glial cell proliferation and migration (Li G.F. et al., 2018), and circ-ANAPC7/miR-181 may participate in acute myeloid leukemia pathogenesis (Chen H. et al., 2018). Other examples of circRNAs functioning as miRNA sponges in cancer processes, such as proliferation, migration, and angiogenesis, are presented in Figure 1 (Mignacca et al., 2016; Liu et al., 2017; Zhong Z. et al., 2017; Dai et al., 2018; Wang H. et al., 2018). In addition, lncRNA (long non-coding RNA), circRNA and mi-RNA interact with each other in a complicated manner, and they combine as RNA networks in cells (Kleaveland et al., 2018). The circRNA/lncRNA/miRNA/mRNA loop is involved in cancer such as bladder cancer (Li M. et al., 2018) due to the complex associations among circRNAs, lncRNAs, miRNA, mRNA and cancer (Nan et al., 2017; Kleaveland et al., 2018).

### Aberrant Transcriptional Regulation or Aberrant RNA Splicing

The patterns of circRNAs in transcriptional regulation in the nucleus may have similarities with some lncRNAs in cancer (Eidem et al., 2016; Schmitt and Chang, 2017). However, the regulatory roles of lncRNAs in transcription are considerably more varied as they accumulate and act in both *cis* and *trans*, whereas circRNAs accumulate and act in *cis* (Chen L.L., 2016). The methods for transcriptional regulation include interaction with Pol II or other associated enzymes (Zhang et al., 2013; Li Z. et al., 2015) and RNA:DNA hybrid formation (Conn et al., 2017). For example, EIcircRNAs such as circEIF3J and circPAIP2 promote the transcription of the host genes through interplay with U1 snRNP, Pol II, and the promoters in HeLa cells and HEK293 cells (Li Z. et al., 2015). However, whether these functions exist in other cancer cells remains unknown. Another study in *Arabidopsis* found that the SEP3 exon 6 circRNA binds to DNA as a R-loop, inhibiting transcription (Conn et al., 2017). Besides these function, circRNAs are involved in RNA splicing via competition with pre-mRNA splicing or as novel small nuclear RNAs (snRNAs) in splicing (Qin et al., 2018). For example, the circularization of circMbl from the second exon of the splicing factor muscleblind competes with canonical pre-mRNA splicing (Ashwal-Fluss et al., 2014). As a downregulated biomarker in non-small cell lung cancer, circ-UBR5 binds *QKI*, *NOVA1*, and *U1* snRNA in the nucleus (Qin et al., 2018).

### Aberrant circRNA-Protein Complexes (circRNPs)

CircRNAs exhibit numerous interactions with a large number of proteins as an RBP decoy or a protein scaffold in the cytoplasm (Du et al., 2016; Schneider et al., 2016; Abdelmohsen et al., 2017; Fang et al., 2018). In breast cancer, circ-Ccnb1 binds H2AX and wild-type p53 to enable p53 wild-type cell survival. However, the p53 mutant generates circ-Ccnb1 to form a complex with H2AX and Bclaf1, ultimately leading to cell death (Fang et al., 2018). Another well-known circular transcript from forkhead box O3 (circ-Foxo3), which is suppressed in breast cancer and non-small cell lung cancer, can bind to some transcription factors (Lu, 2017; Pelletier et al., 2017). The Circ-Foxo3-p21-CDK2 ternary complex inhibits cell cycle progression (Du et al., 2016), and the interaction among Circ-Foxo3, anti-senescent protein ID-1, the transcription factor E2F1 increases in cellular senescence (Du et al., 2017). The binding of circRNAs and proteins associated with translation may lead to unexpected stalling in translation. For example, CircPABPN1 inhibits the binding of PABPN1 mRNA and subsequent translation by competitively binding HuR (Abdelmohsen et al., 2017). Another circRNA derived from the Argonaute (AGO2) gene has the potential to bind HuR as well (Chen Y. et al., 2018). This binding subsequently prevents AGO2 from forming the AGO2-miRNA complex and inhibits gene silencing, which ultimately drives cancer progression (Chen Y. et al., 2018).

### Aberrant Translation

In 2017, circRNAs were first found to be translated under certain conditions (Pamudurti et al., 2017). CircRNA translations can be classified as IRES (internal ribosome entry site) dependent and IRES independent (Tatomer and Wilusz, 2017). IRES-dependent translations are generally found in circ-ZNF609 (Legnini et al., 2017), and IRES-independent translations are generally found in artificial circular RNAs in living HeLa cells (Abe et al., 2015). The aberrant translation of circRNAs can alter tumor malignancy. For example, circ-SHPRH driven by IRES elements can be translated into a tumor suppressor protein, which is associated with patient survival time in glioblastoma (Begum et al., 2018). A circRNA named Circ-FBXW7 can be translated into functional proteins and inhibit glioma tumorigenesis (Yang et al., 2018). Additional research in glioblastoma found that the circular form of the long intergenic non-protein-coding RNA p53-induced transcript (LINC-PINT) could be translated into a peptide, which subsequently inhibits the transcriptional elongation of some oncogenes and thus suppresses the growth of glioblastoma (Zhang et al., 2018). Furthermore, a circRNA named circGpr5 encodes a peptide that interacts with Gprc5a and circGpr5 to promote bladder cancer (Gu et al., 2018).

In addition to disruption of these four functions (that is, as miRNA sponges, in transcription regulation, in protein binding and translation into proteins), circRNAs have the potential to be retro-transcribed and then inserted back into the genome to function as competitive RNA (Dong et al., 2016). Deregulation of circ-Foxo3 and the Foxo3 pseudogene have been detected in tumor growth, and their upregulation has been found to suppress cancer by activating Foxo3 protein (Yang et al., 2016).

## The Aberrant Regulation of Circrnas in Cancer

**FIGURE 2:**
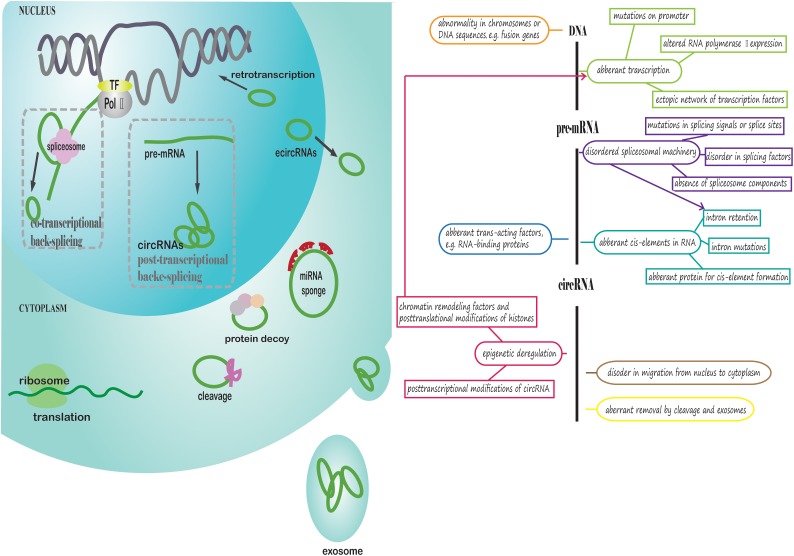
Potential aberrant regulation of circRNA biogenesis, export from the nucleus and removal in cancer cells. The left part of the figure presents how circRNAs are generated, exported from the nucleus to cytoplasm and removed in cancer cells. First, the parental gene sequences of circRNA in cancer may be aberrant. Pre-mRNA is transcribed from DNA, and RNA PolII and transcription factors could be deregulated. CircRNAs are generated through co-transcriptional back-splicing or post-transcriptional back-splicing from pre-mRNA with disordered spliceosomal machinery. EcircRNAs are exported from the nucleus to cytoplasm in a manner similar to linear mRNA. In the cytoplasm, circRNAs may exhibit aberrant functions in cancer. Finally, circRNAs are degraded or exported from the cell through exosomes in an abnormal manner. The right part of figure is a conceptual diagram corresponding to the left side of the figure.

As demonstrated above, aberrant expression of circRNA, i.e., upregulation or downregulation, is prevalent in tumors, which can ultimately promote tumorigenesis or progression. However, why do circRNAs exhibit aberrant expression and function exclusively in cancer cells rather than normal cells? What factors may contribute to circRNA deregulation in cancers? We review and explore answers to these questions in the following section, which is presented in Figure 2.

### Aberrant Events in circRNAs Biogenesis

In normal cells, the accumulation of nascent circRNAs contributes considerably to their detection at steady-state levels (Ashwal-Fluss et al., 2014; Zhang Y. et al., 2016), underlining the importance of circRNA biogenesis. This section is mainly focused on the effect of cancer-related genetic alterations, including single nucleotide variants (SNPs), genomic rearrangements, recurrent somatic mutations, and copy number alterations (Manguso et al., 2018), which modulate the expression of circRNAs through circRNA biogenesis. We discuss aberrant events in circRNA biogenesis in chronological order, and this section is divided into five subsections: aberrant *cis*-elements, aberrant chromosomes and genomes, aberrant transcription, aberrant spliceosomal machinery, and aberrant trans-acting factors.

#### Aberrant *Cis*-Elements

*Cis*-elements typically refer to long complementary flanking introns (repetitive or non-repetitive) in pre-mRNA. *Cis*-elements play a predominant role in the regulation of circRNA production, especially in humans (Ashwal-Fluss et al., 2014). First, as evidence for the intron-driven hypothesis, a species comparative study discovered that short interspersed nuclear elements (SINEs), especially Alu elements, are responsible for robust circRNA production in humans (Dong and Ma, 2017). Remarkably, functions of Alu elements may be involved in their roles as splice acceptors, translation inhibitors and genomic instability inducers and their association with some genetic disorders (Daniel et al., 2015; Kim et al., 2016). In addition to inverted Alu repeat elements (IAREs), miniature intron vectors could induce back-splicing in human genes (Liang and Wilusz, 2014). Second, the high production of fusion circRNAs, which also supports the intron-driven hypothesis. That is because fusion genes generated from the translocations of chromosomes can breed juxtaposition and intron pairing of pre-mRNAs, then the increased intron pairing promotes the production of circRNAs (Guarnerio et al., 2016; Babin et al., 2018). Third, the length of flanking introns has been revealed to be positively correlated with circRNA abundance, which means longer flanking introns drive the generation of circRNAs (Westholm et al., 2014). Thus, if some mutations in complementary sequences render them mismatched and fail to circularize, or if the length of the flanking introns are shortened, circRNA deregulation may occur.

On the other hand, mutations in certain intronic repeats are prevalent in cancers such as gastric cancer (Kim et al., 2013). Intron retention in mature mRNAs is associated with a number of human diseases, including cancer, as an orchestrated phenomenon (Wong et al., 2016). Moreover, intron retention contributes to tumor-suppressor inactivation (Jung and Lee, 2015). In addition, intron retention in the conversion from EIciRNA to ecircRNA not only alters the steady-state levels of circRNAs but also changes their localization because the intronic sequences may function as ribonucleic nuclear retention elements (Chen L.L., 2016).

Similar to intron mutations, editing enzymes have the potential to diminish the complementarity of flanking introns in cancer. For example, ADAR mainly targets Alu elements, and aberrant ADAR activity has been linked to a variety of cancers (Wang et al., 2017). In addition to *cis*-elements, alterations of proteins such as the ribonucleoprotein named HNRNPC, which is related to the formation of Alu elements, should be taken into consideration (Wu et al., 2018).

#### Aberrant Chromosomes and Genomes

Chromosomal and genomic abnormalities such as translocation have been linked to cancers in many studies. First, circRNAs derived from fusion-genes are characteristic of tumors, such as leukemia and non-small cell lung cancer (Guarnerio et al., 2016; Tan et al., 2018). In leukemia, fusion circRNAs promote proliferation and cause therapeutic resistance (Guarnerio et al., 2016). In non-small cell lung cancer, fusion circRNA named F-circEA from the EML4-ALK fusion gene has recently been revealed to promote cell migration and invasion (Tan et al., 2018). The possibility of circularization increases when the chromosomes harbor translocations, which may cause the juxtaposition of intron sequences. In other words, genomic rearrangements generate aberrant *cis*-elements and promote back-splicing (Guarnerio et al., 2016). Chromosomal translocations have the potential to generate cancer-specific circRNAs, the universality of which was confirmed in the models of artificial NPM1-ALK fusion genes (Babin et al., 2018). Second, some circular DNA tumor viruses, such as Epstein–Barr virus (EBV), robustly generate circRNAs in a manner very similar to aberrant genomes given that the viral genome is present in the nucleus of the host cell in an irregular manner (Toptan et al., 2018).

#### Aberrant Transcription

Promoter mutations and aberrant expression or enzymatic activity of RNA PolII can result in transcription suspension (Liu et al., 2013). To better understand the regulation of circRNA transcription, further investigations of conflicts over the order of back-splicing and transcription are required. Although early analysis concluded that splicing events mostly occur co-transcriptionally in most cells and tissues (Pandya-Jones and Black, 2009; Brugiolo et al., 2013), Yang Zhang et al. recently found that the majority of circularizations occur post-transcriptionally (Zhang Y. et al., 2016). The necessity of a functional 3’ end processing signal in back-splicing also supports the post-transcriptional back-splicing (Chen and Yang, 2015). In contrast, the co-transcriptionality of pre-mRNA processing has been confirmed by the fact that splicing and transcription elongation are mutually dependent (Brzyzek and Swiezewski, 2015). The head-to-tail junction reads in the chromatin-bound newly synthesized RNA, and the competition between linear splicing and back-splicing support co-transcriptionality as well (Ashwal-Fluss et al., 2014); however, we remain skeptical, and in an alternative study, we have been able to potentially determine whether this event occurs post-transcriptionally or co-transcriptionally based on the length of the flanking intronic repeats: long intronic repeats are more likely to promote co-transcriptional back-splicing (Kramer et al., 2015). Co-transcriptional and post-transcriptional splicing facilitates different methods of regulation. If back-splicing occurs co-transcriptionally, the efficiency of back-splicing is strongly influenced by the transcription elongation rate. For example, Pol II mutants in R749H or E1126G have the capacity to slow down or speed up transcription and circularization, respectively (Zhang Y. et al., 2016).

Transcription factors, which are vital players in transcription, are associated with various tumor-specific genes (Atkins et al., 2016) and circRNAs. For example, the oncogenic transcription factor c-Myc regulates the expression of numerous circRNAs by binding to the promoter regions of parental genes. These Myc-regulated circRNAs are important in cell proliferation via the Ras signaling pathway in cancer (Gou et al., 2017). Moreover, transcription factors were confirmed as regulators of miRNAs in tumorigenesis and progression, and circRNAs and miRNA sponges may also participate in these processes. For example, the impact of Myc on the circRNAs/miRNAs axis has also been reported (Gou et al., 2017).

#### Aberrant Spliceosomal Machinery

Canonical spliceosomal splicing mechanism and back-splicing mechanism are involved in the biogenesis of circRNA (Quan and Li, 2018). The back-splicing mechanism is affected by canonical splicing signals (Starke et al., 2015). Given that aberrant RNA splicing has been linked to cancer (Scotti and Swanson, 2016), the spliceosomal machinery may contribute to circRNA deregulation in cancer. Mutations in splice sites and spliceosome components, including five small nuclear RNAs (snRNA), affect the steady-state levels of circular RNAs (Liang et al., 2017).

First, recurrent mutations in spliceosomal genes, such as SF3B1, SRSF2 and U2AF1, are responsible for mis-splicing and vulnerabilities in cancer (Chabot and Shkreta, 2016; Dvinge et al., 2016). Second, splicing factors (hnRNPs, SR proteins) increase Laccase2 circular RNA levels in conjunction with intronic repeats (Kramer et al., 2015). The frequent deregulation of SR/hnRNP proteins induces apoptotic gene dysfunction in cancers (Kedzierska and Piekielko-Witkowska, 2017). Third, dozens of splicing factor genes are differentially expressed in cancer (Sveen et al., 2016). Alternative RNA splicing events, which are diverse in the biogenesis of circRNA, also characterize cancer (Tremblay et al., 2016; Zhang X.O. et al., 2016).

#### Aberrant Trans-Acting Factors

Trans-acting factors are also important triggers of back-splicing in addition to *cis*-acting factors. To date, Mbl and QKI are the most typical trans-acting factors as revealed in current studies. Mbl binds to the flanking introns of circMbl in *Drosophila* and human (Ashwal-Fluss et al., 2014). QKI, which is regulated during the human epithelial-mesenchymal transition (EMT), binds to circRNAs in a manner quite similar to Mbl (Conn et al., 2015). Methylation of the QKI promoter, which reduces QKI expression, may be critical in colorectal cancer (Darbelli and Richard, 2016; Iwata et al., 2017). In addition, QKI also inhibits aberrant splicing QKI (Zong F.Y. et al., 2014), and these actions may collectively result in aberrant circRNA expression.

Some enzymes, such as RNA helicase, are vital players in regulating circRNA in cancer. The RNA helicase DHX9 is overexpressed in lung cancer (Cao et al., 2017), and its downregulation reduces the number of cancer cells (Lee et al., 2016). DHX9 reduces the expression of circRNAs, such as cSMARCA5, by directly binding to Alu elements and regulating circRNA-producing genes, RNA processing and translation (Yu et al., 2018). In addition, DHX9 interacts with the editing enzyme ADAR given that co-depletion of ADAR and DHX9 increases circular RNA production (Aktas et al., 2017).

Other proteins with the potential to function as trans-acting factors in circRNA biogenesis include the immune factors NF90/NF110 (Li et al., 2017). These proteins increase circRNA expression in a manner similar to chromosome translocations (juxtaposing and intron pairing) and serve as components of circRNPs in the antiviral immune response in HeLa cells (Li et al., 2017).

### Aberrant Epigenetic Regulation

Advanced sequencing has revealed that greater than 50% of cancers exhibit mutations involved in chromatin organization (Kleppe et al., 2018).

There are two hypothetical mechanisms behind epigenetic aberrations involved in circRNA deregulation. First, chromatin remodeling factors and post-translational modifications of histones impact the transcription rate, which may subsequently affect the production of circRNAs (Zhang Y. et al., 2016). Second, chromatin remodeling is likely to affect diverse alternative splicing events involved in the biogenesis of circRNAs (Chen and Yang, 2015). For example, promoter CpG island hypermethylation-associated silencing of some genes, such as TUSC3 (tumor suppressor candidate 3) and POMT1 (protein O-mannosyltransferase 1), reduce circRNA production in cancer (Ferreira et al., 2018).

In addition to DNA methylation and histone modifications, post-transcriptional modifications of circRNAs are associated with circRNA deregulation. The three most abundant epitranscriptomic marks of RNA are pseudouridine (Ψ), N^6^-methyladenosine (m^6^A) and 5-methylcytosine (m^5^C). These marks tend to determine the fates of long noncoding RNAs. RNA modifications promote colorectal cancer by upregulating oncogenes or downregulating tumor suppressor genes (Porcellini et al., 2018). Cancer-related long noncoding RNAs, such as MALAT1, exhibit multiple post-transcriptional modifications; however, no aberrant modification in circRNA has been discovered (Jacob et al., 2017). Among these RNA modifications, m^6^A is the most common modification (Dominissini et al., 2012). Of note, m^6^A is rich in circRNA and drives translation initiation (Molinie et al., 2016; Nan et al., 2017; Yang et al., 2017).

### Aberrant Regulation in circRNA Export From Nucleus

Upon formation in the nucleus, ecircRNAs tend to be transported to the cytoplasm via a mechanism similar to linear RNA export, and the nuclear pore complex is an important player (Hautbergue, 2017). A methyl-guanosine cap and poly(A) tail are the determinants for RNA exportation from the nucleus (Tuck and Tollervey, 2013). The precise mechanism by which circRNAs without free ends are exported from the nuclear pore complex remains unknown. However, recent studies have found that the length of mature circRNAs plays an important role in determining whether the circRNA is exported or retained, which contradicts the retained intron restriction hypothesis (Huang et al., 2018; Wan and Hopper, 2018). By knocking out the genes associated with RNA exportation, UAP56 and URH49 have been identified to control the location of circRNAs in HeLa cells. In detail, UAP56 is responsible for the export of long circRNAs, whereas URH49 is responsible for the short circRNAs (Huang et al., 2018; Wan and Hopper, 2018). However, this research is based on artificial circular RNAs in HeLa cells, and the mechanism by which natural circRNAs with known functions are exported from the nucleus requires further exploration. Although studies on circRNA export are limited, different cellular localizations of non-coding RNAs have been linked to numerous diseases (Tuck and Tollervey, 2013). It is hypothesized that dysfunctions in the “transporting” or “sorting” mechanisms of circRNAs may contribute to aberrant circRNA expression in tumors (Chen and Shan, 2015). RNA binding proteins as protein cargos may be involved in circRNA migration. Given that lncRNA subcellular fates are determined by nuclear retention signals and the long non-coding ribonucleoproteins complex (Chen L.L., 2016), some disorders in location signals and protein traps might account for circRNA deregulation in cancer. In addition, epigenetic features, such as chromosome structure, could affect the localizations of lncRNAs because high-order chromosomes that form a loop may exhibit increased possibilities for nuclear retention of lncRNAs (Tuck and Tollervey, 2013).

### Aberrant circRNA Removal by Cleavage and Exosomes

Degradation of circRNAs in the cytoplasm remains largely uncharacterized, but there is evidence for their existence. For instance, AGO2/miR-671-mediated cleavage of CiRS-7 autoregulates CiRS-7 as confirmed in HEK293 and HeLa cells (Hansen et al., 2011). Thus, the anomalous expression of miR-671 and AGO2, the major components of the RNA-induced silencing complex (RISC), influence the amount of CiRS-7.

Packaging and export of circRNAs by extracellular vesicles or microvesicle release seems common in mammalian cells (Lasda and Parker, 2016) given that circRNAs have been noted in exosomes and blood plasma (Li Y. et al., 2015). Accordingly, alterations of proteins associated with the packaging of extracellular vesicles or microvesicles and their protein compositions could cause deregulation. Additionally, extracellular vesicles could influence the tumor microenvironment by communicating with other cells (Wu et al., 2017), and significantly impact the immune response in tumor cells. Aberrant extracellular vesicles have been recognized as emerging therapeutic targets for cancer (Wu et al., 2017).

## Conclusion

CircRNAs that are aberrantly expressed in cancers exhibit abnormal roles as miRNA sponges, protein decoys, transcription regulators, or regulators of translation into proteins. The potential mechanisms involved in deregulation were outlined, including in their biogenesis from parental genes, export from the nucleus to the cytoplasm and removal from the cell.

The underlying mechanisms are potentially considerably more complicated than that described above, as they may exhibit multiple interactions. For instance, intron retention that contributes to the aberrant *cis*-elements may result from the dysfunctions of spliceosomal machinery, such as splice site mutations (Ge and Porse, 2014).

As a cancer cell is viewed as the outcome of alterations in genetics, epigenetics and epitranscriptomics (Porcellini et al., 2018), the deregulation of circRNAs may be associated with these factors as well as other uncharacterized components. The deregulation mechanism of circRNAs is a new field that requires further exploration. In the future, more deregulated circRNAs will be discovered in human diseases, especially cancer, and circRNAs may display more functions. The profound understanding of the deregulation of circRNA mechanisms may provide more possibilities for better diagnosis, prognosis, and treatment of cancer. To date, the potential advantages of circRNAs as biomarkers for tumors have been highlighted given their abundance, stability and tissue-specific expression (Qian et al., 2018). Specific circRNAs can be detected in the blood plasma of patients to track the progression of the corresponding cancer. A better understanding of the mechanisms involved will serve as a significant breakthrough in this area.

## Availability of Data and Material

The material supporting the conclusion of this review has been included within the article.

## Author Contributions

All authors read and approved the final manuscript.

## Conflict of Interest Statement

The authors declare that the research was conducted in the absence of any commercial or financial relationships that could be construed as a potential conflict of interest.

## Abbreviations

circRNA: circular RNA
Mbl: muscleblind
m^6^A: N^6^-methyladenosine
QKI: quaking
